# Experimental study on the shear creep behavior of residual soil with varying rock content

**DOI:** 10.1371/journal.pone.0311156

**Published:** 2025-02-13

**Authors:** Chuang Wang, Jinyu Dong, Shengwen Qi, Jiancang Zhao, Lei Xue, Dengpo Fan

**Affiliations:** 1 College of Geosciences and Engineering, North China University of Water Resources and Electric Power, Zhengzhou, China; 2 Key Laboratory of Shale Gas and Geoengineering, Institute of Geology and Geophysics, Chinese Academy of Sciences, Beijing, China; 3 Innovation Academy for Earth Science, Chinese Academy of Sciences, Beijing, China; 4 Henan Water Conservancy Survey Company Limited, Zhengzhou, China; Guizhou University, CHINA

## Abstract

Using residual soil from the Shanghecun landslide in the western Henan Province, shear creep tests of residual soil samples with different rock contents (RCs) were performed to explore the creep characteristics, creep rate, and long-term strength of the residual soil. The test results indicate that the residual soil samples with different RCs display typical creep characteristics. With increasing RC, both the instantaneous deformation and the total creep deformation of the residual soil gradually decrease. The shear strain of the residual soil increases gradually with increasing shear stress for the different RCs. With increasing time, the slope of the isochronous stress–strain curves of the residual soil samples with different RCs increases gradually. The Burgers model can simulate the rheological process of the residual soil samples with different RCs. The RC has a significant effect on the shear strength and the long-term strength of the residual soil. With increasing RC, the shear strength and the long-term strength of the residual soil gradually increase, with the long-term strength being approximately 39%–63% of the shear strength.

## 1. Introduction

Residual soil is a type of soil–rock mixed discrete material with a wide particle size distribution formed by rock weathering. Because of the complex physical and mechanical properties of soil–rock mixtures, which are different from soil and rock masses, the problem of slope deformation and failure caused by the long-term action of an upper load or seepage conditions is complicated [1–5]. The essence of this problem consists of both settlement in the vertical direction and instability caused by shear deformation [6–9]. Because of its long-term effects, shear creep has become a focus of research, especially studies of the characteristics of shear creep.

Existing research results [10, 11] show that creep properties and aging characteristics are inherent mechanical properties of geotechnical materials and that even hard rocks such as marble and granite have creep characteristics. Among the various creep properties of rock and soil materials, the long-term strength of these materials is of great significance to the stability of a slope. Many engineering practices and studies show that the instability and failure of rock and soil are closely related to time. A large number of engineering practices show that the strength parameters of rock and soil masses change with time, that is, there is a time effect. However, there are few research results concerning the long-term strength parameters of rock–soil masses with time. Therefore, it is necessary to study the shear creep characteristics of residual soil samples with different rock contents (RCs) using experimental methods.

The shear creep characteristics of rock and soil materials play an important role in controlling the long-term stability of large-scale slopes, dam foundations, and other major projects, and relevant researchers have performed a large amount of experimental research on this topic. Yan et al. [12] conducted shear creep tests on soil in the slip zone of the Dayantang landslide under different consolidation pressures, obtained creep curves of the pressure and shear forces at all levels, and found that the soil in the slip zone showed obvious rheological characteristics. Li et al. [13] studied the creep characteristics of an undisturbed shear zone using a triaxial creep test and found that the inhomogeneity of the undisturbed soil samples resulted in creep characteristics that differed from the ideal theoretical model. Tang et al. [14] and Zhu et al. [15] studied the creep characteristics of loess and slip zone soil, respectively, under different water content conditions using a series of direct shear creep tests. Qin et al. [16] and Luo et al. [17] studied the creep characteristics of rock under changing reservoir water level conditions, discussed the mesoscopic damage mechanism of rock under the action of a water-loss cycle, and quantitatively described the amount of rock damage. Liu et al. [18] found that the settlement of an embankment filled with a soil–rock mixture obeyed a nonlinear creep law and proposed a modified Kelvin creep model to account for the nonlinear viscoelastic deformation of the material. Yao et al. [19] conducted triaxial creep tests for a soft soil at different stress levels, verified the performance of the soft-soil creep model, and obtained an attenuation function of the cohesion. Wen and Jiang [20] conducted a series of shear creep tests on gravel-bearing clay and studied the influence of the gravel content on the creep behaviour of the material. Yuan et al. [21] used an improved direct shear creep instrument to conduct shear creep tests on sand samples with different particle compositions and water contents and studied the effects of the particle composition and material composition on the characteristics of sandy-soil shear creep. Wang et al. [22, 23] verified the motion mode of a landslide in the Three Gorges Reservoir area using an in-situ direct shear creep test and an indoor annular shear creep test and found that the soil in the shear zone exhibited a non-attenuation creep phenomenon.

The above research results provide a good reference for studies of the creep characteristics of residual soil, especially with respect to the research methods and the establishment of creep models. However, the results pertaining to the shear creep law of residual soil samples with different RCs have not been reported. This paper, based on laboratory results, attempts to clarify the effect of rock contents on creep behavior of residual soil.

In this paper, the residual soil of the Shanghecun landslide in the western Henan Province is taken as the research object. Via indoor shear creep tests of residual soil samples with different RCs, this paper systematically examines the influence of the RC on the creep characteristics and the long-term strength of the residual soil. The results obtained in this paper provide a reference for engineering designs and are significant with respect to further revealing the time-effect law of residual soil and related theoretical research.

## 2. Shear creep test of residual soil

### 2.1 Basic characteristics of residual soil

**Fig 1:**
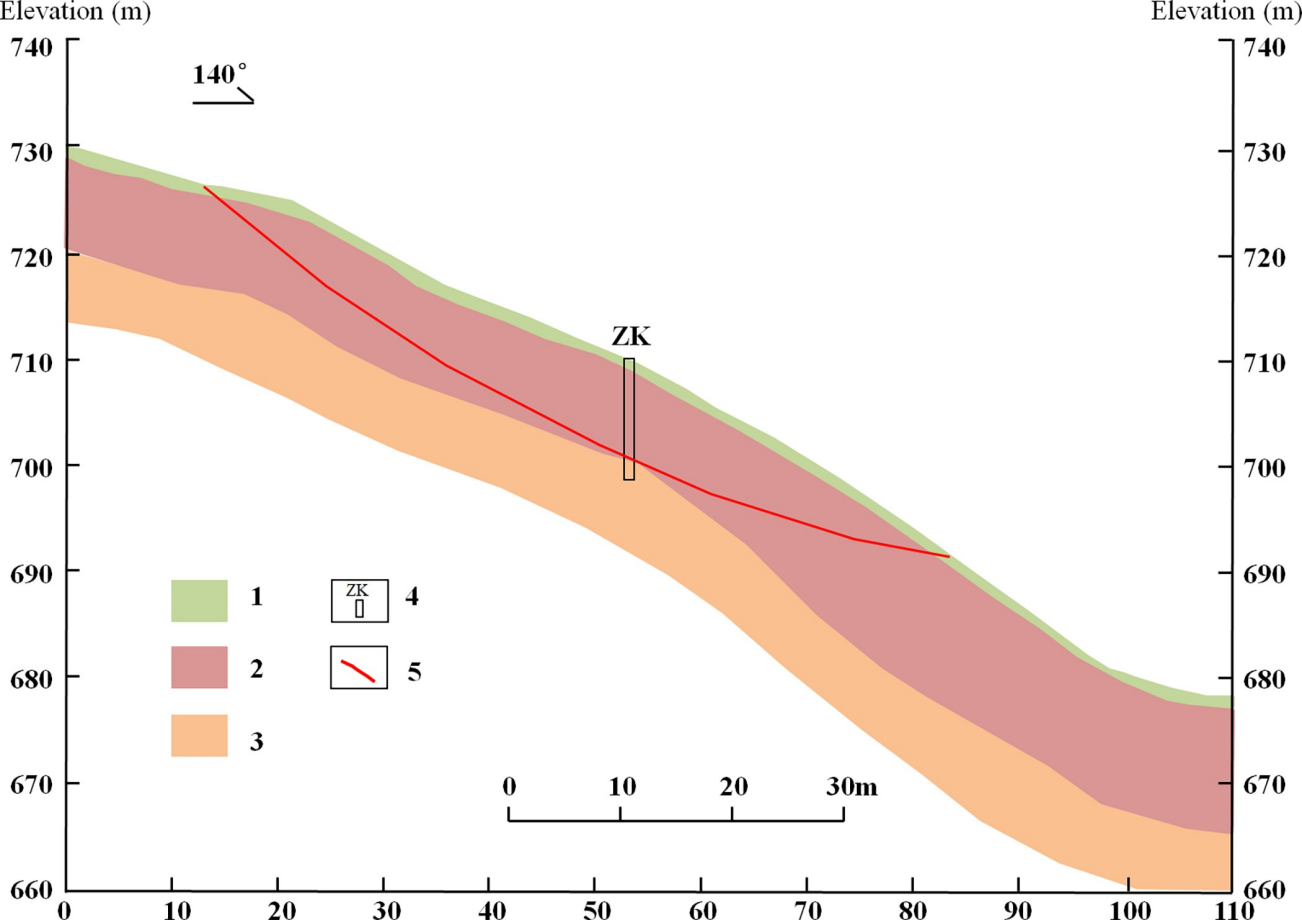
Simplified cross section of the Shanghecun landslide (1: gravel soil; 2: gravel soil mixed with silty clay; 3: mica-quartzose schist; 4: borehole; and 5: slip surface).

**Fig 2:**
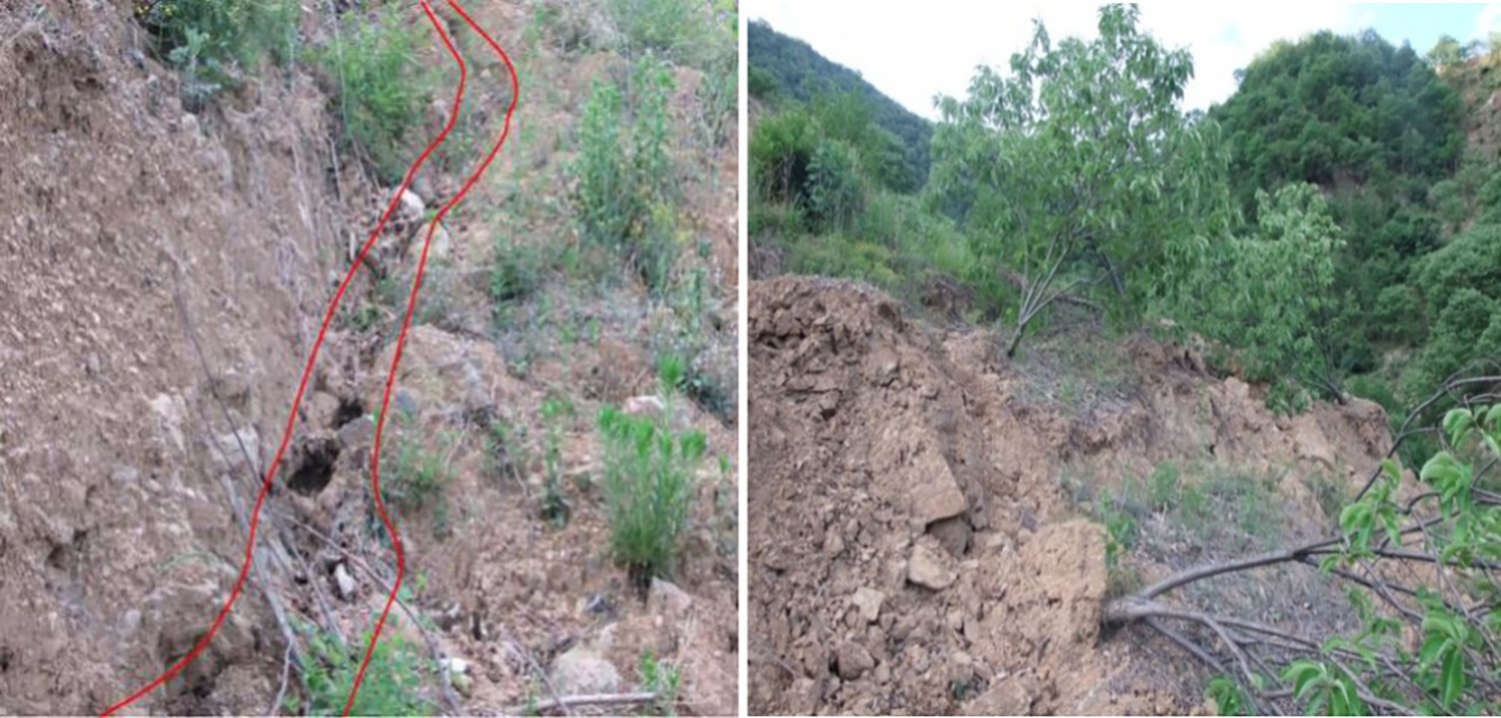
Signs of slow-sliding slope deformation. (a) Tensile crack, (b) Fallen trees.

The tested residual soil was taken from the Shanghecun landslide in the western Henan Province. The perimeter of the landslide is clear and semi-elliptical on the plane, and the sliding body is narrow at the top and wide at the bottom. The length of the landslide is approximately 127 m, and the width of the front edge is approximately 103 m. The thickness of the sliding body is 6–12 m, with an average thickness of approximately 8.5 m (Fig 1) and a volume of 6.5 × 10^4^ m^3^. The slow movement of the landslide is manifested by cracking through the top of the slope, swelling of the ground in the middle and lower part of the slope, and a serious inclination of the trees in the landslide area (Fig 2). The top of the Shanghecun landslide and the base rock in the central area are exposed, and the entire landslide area is covered by Quaternary residual soil and colluvial soil. According to the field geological survey data, combined with geotechnical laboratory tests, the landslide is thought to be Quaternary residual soil consisting of block rock soil mixed with silty clay. The block rock is primarily quartz schist, with a block diameter of approximately 20–45 cm and a light gray color. The location of block rock soil differs, as well as the content of the block rock, with the content of block rock being approximately 45%–75%. The surface vegetation on the slope is lush, and the block rock soil is rich in plant roots. The soil is silty clay and light gray, with bluish gray stripes. In its natural state, the density of the residual soil is 2.03 g/cm^3^ and the water content is 15%.

The maximum controlled particle size in the experiment was 80 mm, and particles larger than 80 mm were replaced by an equivalent amount of particles with sizes ranging from 2 mm to 80 mm [24–26]. According to the Code for Investigation of Geotechnical Engineering [27], 2 mm is defined as the boundary particle size between soil and rock. The percentage of the mass of block stones between 2 mm and 80 mm to the total mass of residual slope soil is defined as the “rock content” [25, 26]. Indoor particle size distribution tests were performed on the samples in their natural state, and the test results are shown in Table 1. It can be seen that there is a high content of block stones in the residual soil. The proportion of particles with a diameter of 2–80 mm is as high as 85% (i.e., the RC is 85%), and the proportion of fine particles with a diameter of less than 0.075 mm is only 0.6%. According to the calculation, the non-uniformity coefficient *Cu* = 9 > 5 and the curvature coefficient *Cc* = 2.3, indicating that the residual soil samples had good gradation.

**Table 1:** Particle size content of the residual soil samples.

Grain size/mm	0.075	1	2	5	10	20	40	60	80
Percent finer by weight/%	0.60	7.40	14.20	25.00	50.10	66.10	75.50	87.80	100

### 2.2 Test equipment and procedure

**Fig 3:**
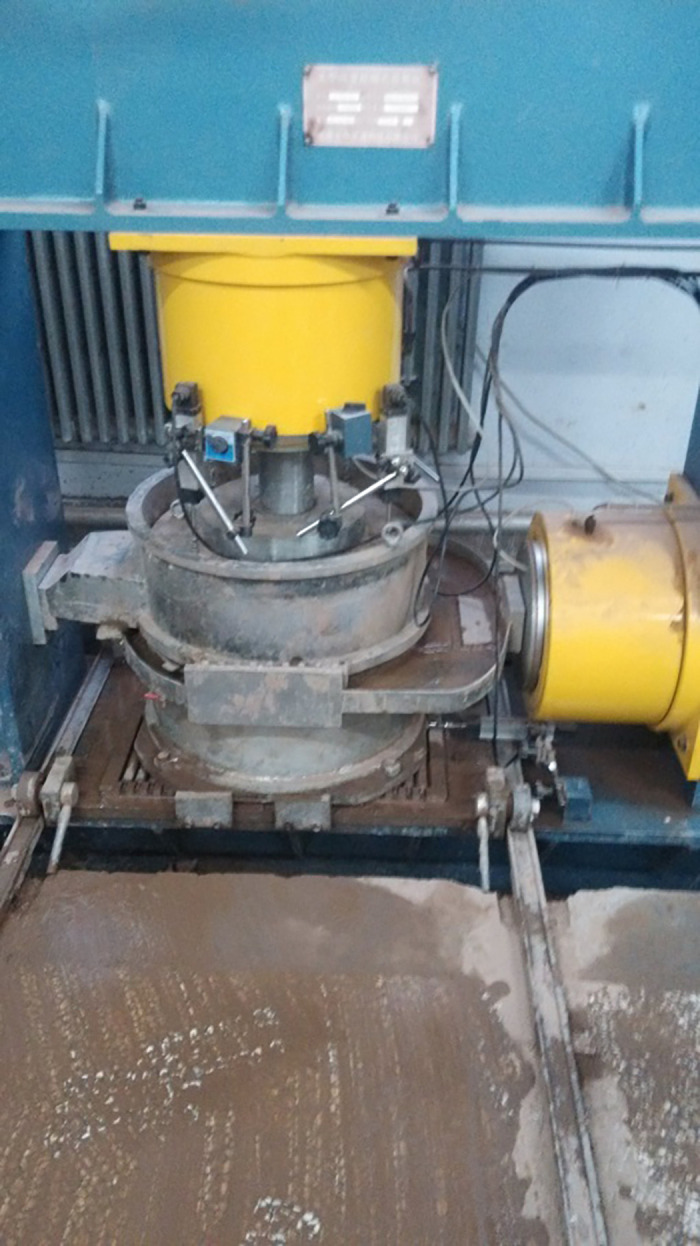
Large direct shear apparatus.

**Fig 4:**
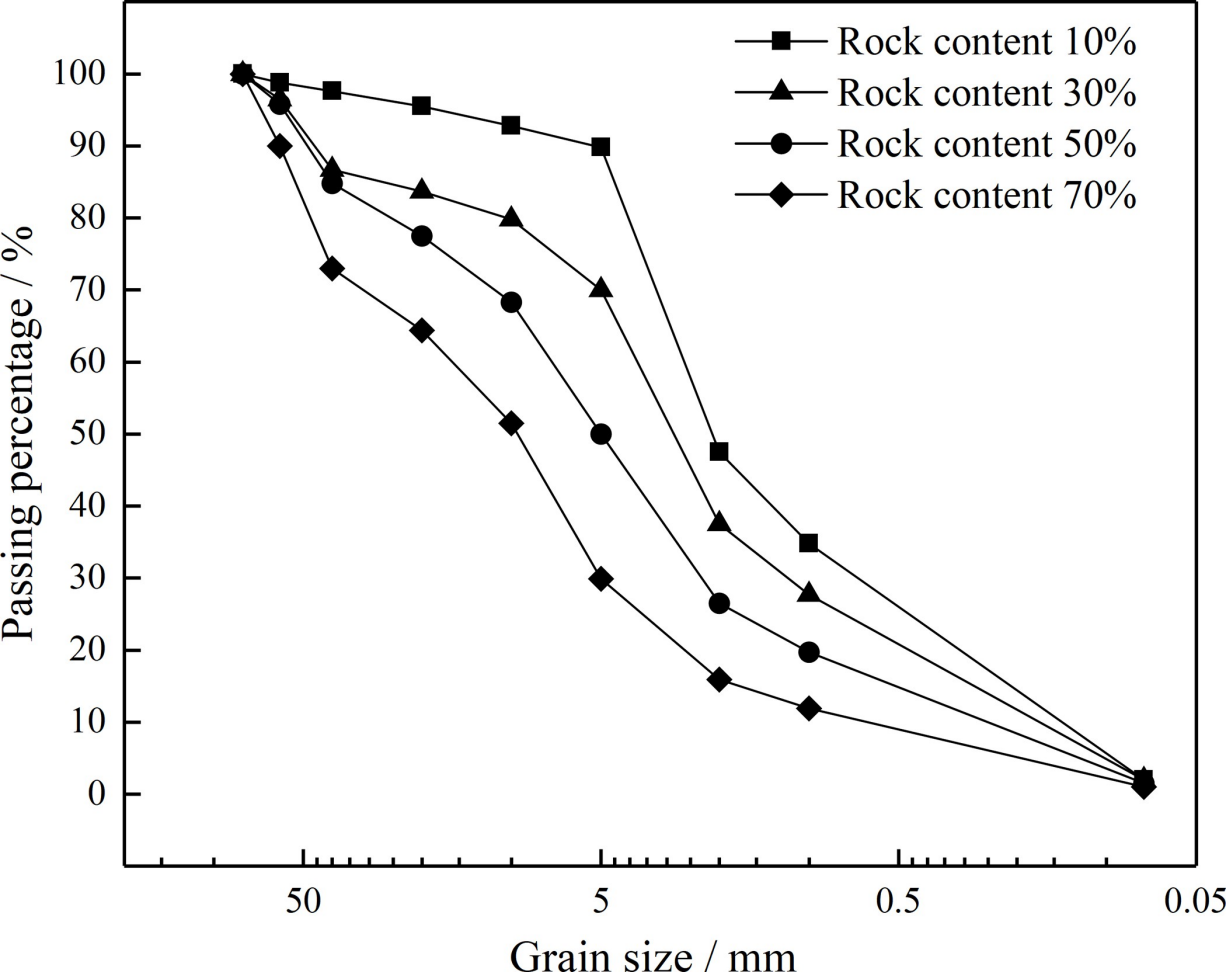
Particle size distributions of the residual soil samples with different RCs.

According to the basic characteristics of the residual soil, to further study the influence of the RC on practical engineering, samples with block RCs of 10%, 30%, 50%, and 70% were prepared with the water content of the samples being 15%. A ZJ50-2G large-scale direct shear test machine from the College of Geosciences and Engineering, North China University of Water Resources and Electric Power was used to perform the direct shear creep tests. Data were automatically collected during the test, primarily by one horizontal displacement sensor and four vertical displacement sensors, as shown in Fig 3. Indoor large-scale direct shear creep tests were performed on the remolded samples. The gradation curves of the residual soil samples with different RCs are shown in Fig 4. The vertical consolidation pressure applied during the tests was 200 kPa. When the vertical displacement of a sample was less than 0.05 mm within 1 h, the sample was assumed to have reached consolidation stability [28]. The classification of the shear stress in the creep test was determined according to the strength parameters of the residual soil samples with different RCs as measured via a conventional direct shear test. The stress loading control mode was adopted for the shear creep test process. When the shear deformation was less than 0.01 mm/d, the next level of the shear load was applied [29]. The shear strength and the shear creep test scheme of the residual soil samples with different RCs are shown in Table 2. The temperature and humidity were kept constant during all of the tests.

**Table 2:** Conditions of the shear creep tests.

Consolidation pressure/kPa	Rock content/%	Shear strength/kPa	Shear stress/kPa
200	10	140	28, 56, 84, 112
200	30	161.5	32, 64, 96, 128
200	50	184	36, 72, 108, 144
200	70	198	40, 80, 120, 160

## 3. Test results

### 3.1 Shear creep characteristics

**Fig 5:**
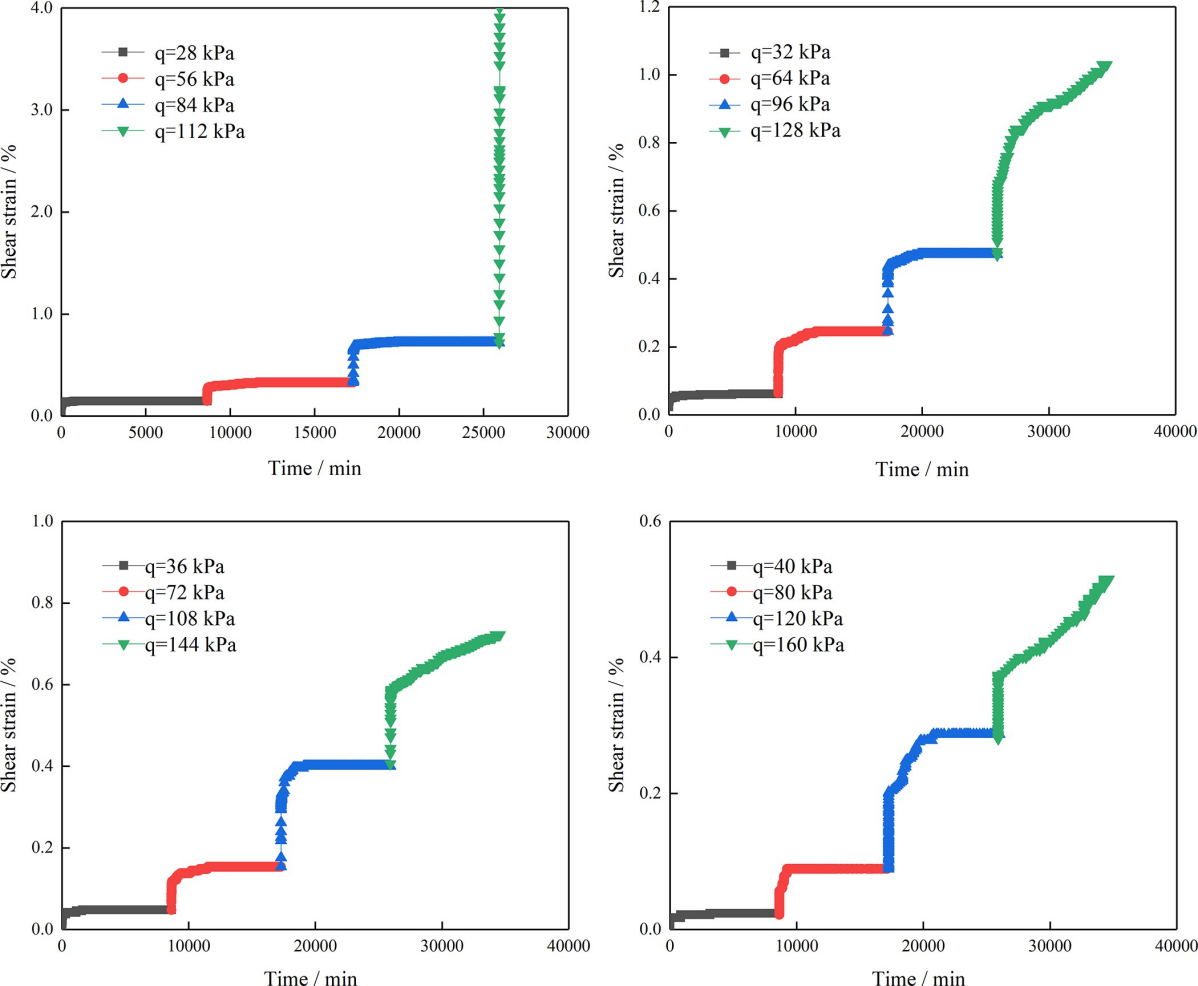
Shear strain–time curves of the residual soil. (a) RC = 10%, (b) RC = 30%, (c) RC = 50%, (d) RC = 70%.

Fig 5 shows the shear creep curve of the residual soil under different shear stresses. The test results indicate that the residual soil is a typical creep material and that it exhibited the following deformation and failure characteristics during the creep test. (1) Under a constant normal load, after the action of shear stress at all levels, the samples first showed characteristics of instantaneous deformation and a large shear strain appeared in a short time. In general, instantaneous deformation accounts for the main part of the total deformation and, with increasing RC, the instantaneous deformation of the samples decreased gradually. (2) With the passage of time, the rate of increase of the deformation changed gradually from fast to slow and finally reached a stable rheological rate. (3) When the applied stress level was high, if the deformation increased sharply, the stable creep stage almost did not exist; instead, accelerated creep occurred directly and the samples were damaged in a short time, as shown in Fig 5(A). (4) Under a constant normal load, the shear strain generally changed corresponding to each level of shear stress applied, as shown in Fig 5(A)–5(D), and as the shear stress increased incrementally, the shear strain also increased.

**Fig 6:**
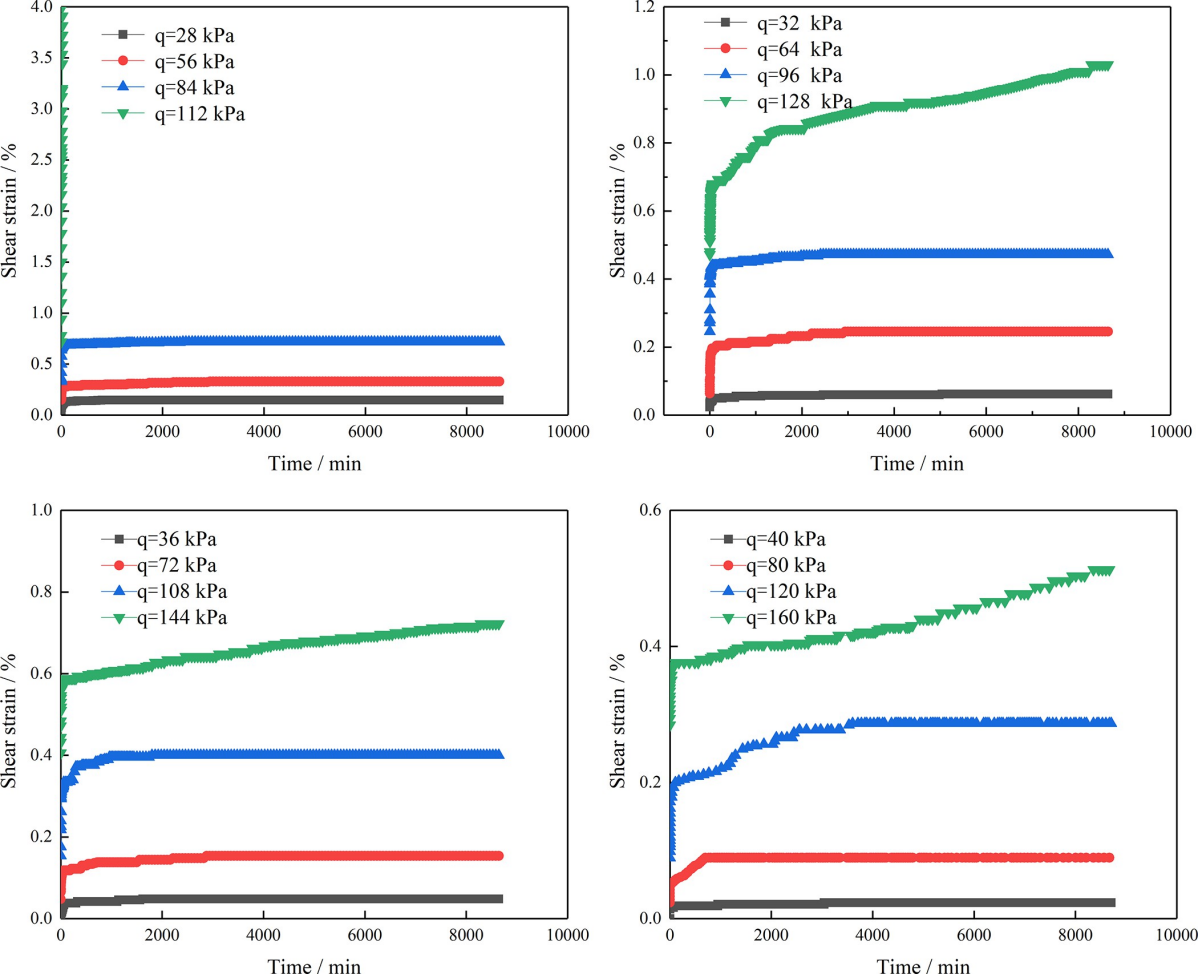
Shear strain–time curves according to the Boltzmann superposition principle. (a) RC = 10%, (b) RC = 30%, (c) RC = 50%, (d) RC = 70%.

At the same time, it can be seen from Fig 6 that the creep curves of the residual soil samples with different RCs belong to the class of unstable creep curves and that the creep process roughly presents three creep forms. (1) The first is attenuated creep. In the initial stage, when each level of the load is applied to the sample, the deformation of the sample, which is primarily composed of elastic and plastic deformation, increases sharply and the creep deformation rate is large. This indicates that the position of the soil and rock in the sample is constantly adjusted when the sample is subjected to shear stress. As time goes by, the soil and rock particles gradually become denser and the creep rate is continuously decreasing and then tends to a stable value, showing attenuated creep. (2) The next form of creep is constant velocity creep. When the soil–rock mixture is under constant stress, the position between the internal particles gradually becomes stable. The compactness increases continuously, and the strain increases slowly; therefore, the creep rate gradually tends to a stable value with increasing time. At this time, the soil–rock mixture enters the constant velocity creep stage. (3) Finally, accelerated creep occurs. As the time of the load acting on the soil–rock mixture continues to increase, the creep deformation of the sample also continues to increase. When the shear stress applied to the soil increases sharply, the creep of the soil reaches its maximum value, which eventually leads to shear failure of the soil as a result of excessive deformation.

Using the Boltzmann superposition principle [30–32] to process the original data, the shear strain and time creep curves under various shear stresses can be obtained. Fig 6 shows curves of the shear strain versus time using Boltzmann superposition processing based on Fig 5.

The following can be concluded from the test results. (1) For the four sample sets with different RCs under the same consolidation pressure of 200 kPa, during the staged loading process, there is a large instantaneous deformation under each level of shear stress. The deformation of the samples in the first hour at each level of load accounted for more than 60% of the total deformation, and the instantaneous deformation increased with increasing shear stress. (2) In the creep test, the creep deformation of the samples is closely related to the shear stress. When the shear stress acting on the sample increases, the creep deformation is positively related to the shear stress. As the creep deformation of the sample increases, the final sample cannot easily enter stable creep and the time to reach stable creep increases. (3) Even under a small shear stress, the deformation of the sample increases with time, which clearly indicates that the soil has obvious creep characteristics. (4) The test results indicate that the four groups of soil–stone samples with different RCs are under the action of the fourth-level shear stress and that the creep deformation of the samples increases with time, showing accelerated creep. In particular, the samples with a 10% RC rapidly enter accelerated creep under a shear stress 0.8 times the shear strength and the samples show shear failure. (5) It is obvious from the displayed creep curves that the RC has a significant influence on the creep of the residual soil. The creep deformation of the residual soil decreases with increasing RC. This is due to the poor roundness of the stones; with increasing RC, the friction between the stones increases, resulting in the shear strength of the mixture being improved, such that the ability of the sample to resist shear deformation also improves. (6) Under the same consolidation pressure, when shear stresses of 0.2 and 0.4 times are applied to the four groups of samples, the creep deformation of the residual soil samples with different RCs is relatively small and the growth of the creep deformation is relatively slow. When the shear stress is 0.6 times of shear strength, the creep deformation and creep deformation rate of the samples increase. Meanwhile, under a shear stress 0.8 times the shear strength, the residual soil samples with different RCs enter the accelerated creep stage. It can be seen that the shear strength of a landslide plays an important role in controlling its stability.

### 3.2 Analysis of isochronous stress–strain curves

**Fig 7:**
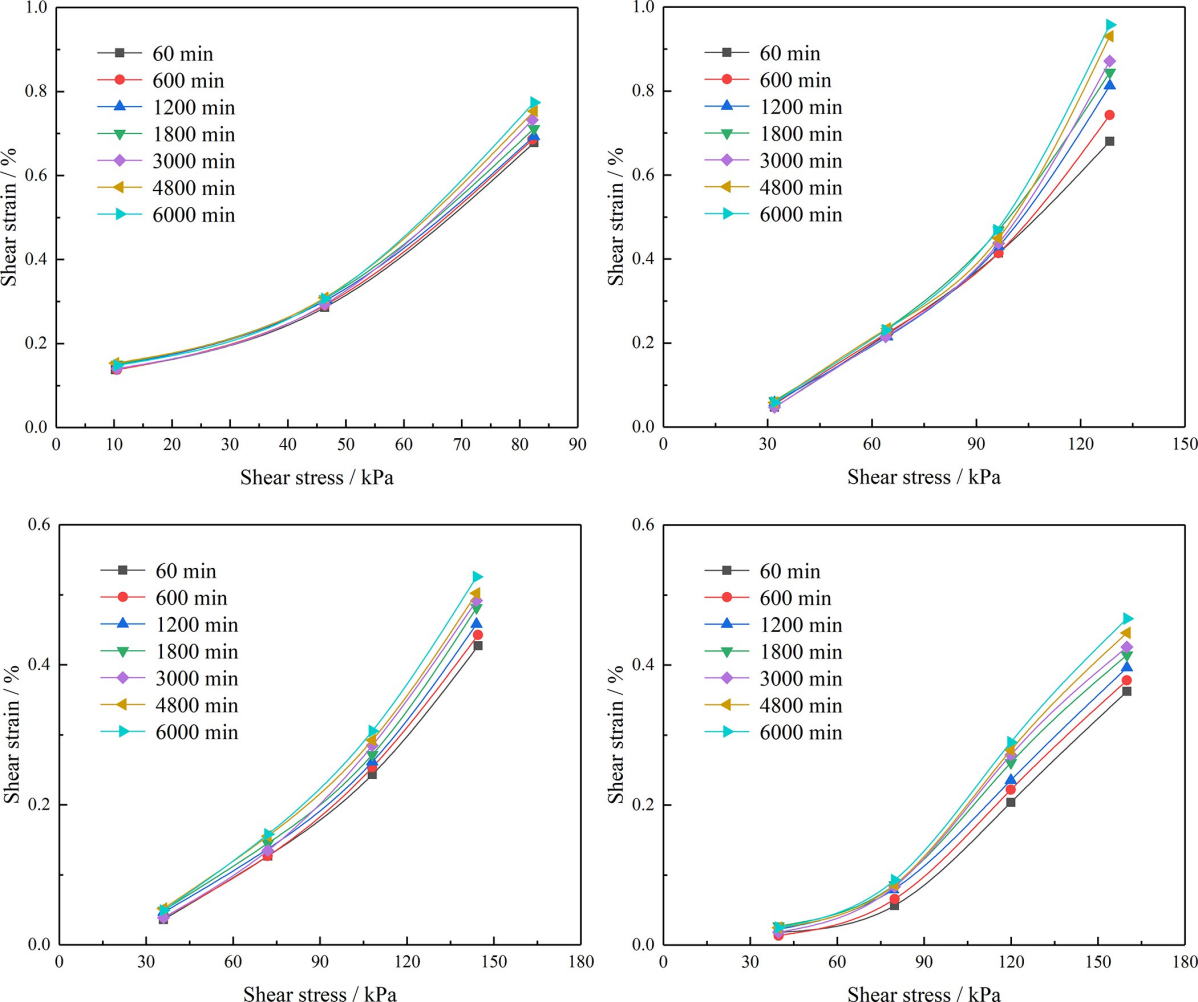
Isochronous stress–strain curves of the residual soil samples. (a) RC = 10%, (b) RC = 30%, (c) RC = 50%, (d) RC = 70%.

According to the test data, the creep curves of the four sets of samples with different RCs were obtained and the strain at seven different time points corresponding to each sample under its respective shear stress was extracted to construct isochronous stress–strain curves, as shown in Fig 7. Because the samples with 10% RC were rapidly damaged under the action of the fourth-order shear load, only the isochronous stress–strain curves of the first third-order load could be made.

The following can be concluded from the test results. (1) The isochronous stress–strain curves of the residual soil samples are nonlinear because of their mixture of soil and rock. When the shear stress increases, the slope of the curve becomes larger and the deformation increases. The nonlinear creep characteristics of the residual soil therefore need to be considered when establishing a creep constitutive model. (2) With increasing time, the slope of the isochronous stress–strain curves of the residual soil samples with different RCs gradually increases, indicating that the creep increment of the soil at the same creep time is increasing, which is a manifestation of decreasing soil strength. (3) The creep deformation of the soil decreases with increasing RC. This is due to the poor roundness of the stones in the residual soil. With increasing RC, the friction between the rock and soil particles becomes increasingly larger, the strength of the soil gradually increases, and the ability of the mixture to resist deformation becomes increasingly enhanced. (4) When the specimens are subjected to small shear stress, the creep strain increases only a little, the stress–strain curves show a roughly linear correlation, and the soil experiences attenuated creep deformation. When the shear stress increases to 0.6–0.8 times the shear strength, the strain growth rate of the soil increases gradually, the soil creep increases exponentially, and the soil enters the constant or accelerated creep state. (5) Under different shear stresses, the isochronous stress–strain curves of the residual soil samples show various stress–strain curves, showing a trend of normalization.

## 4. Shear creep model of the residual soil

**Fig 8:**
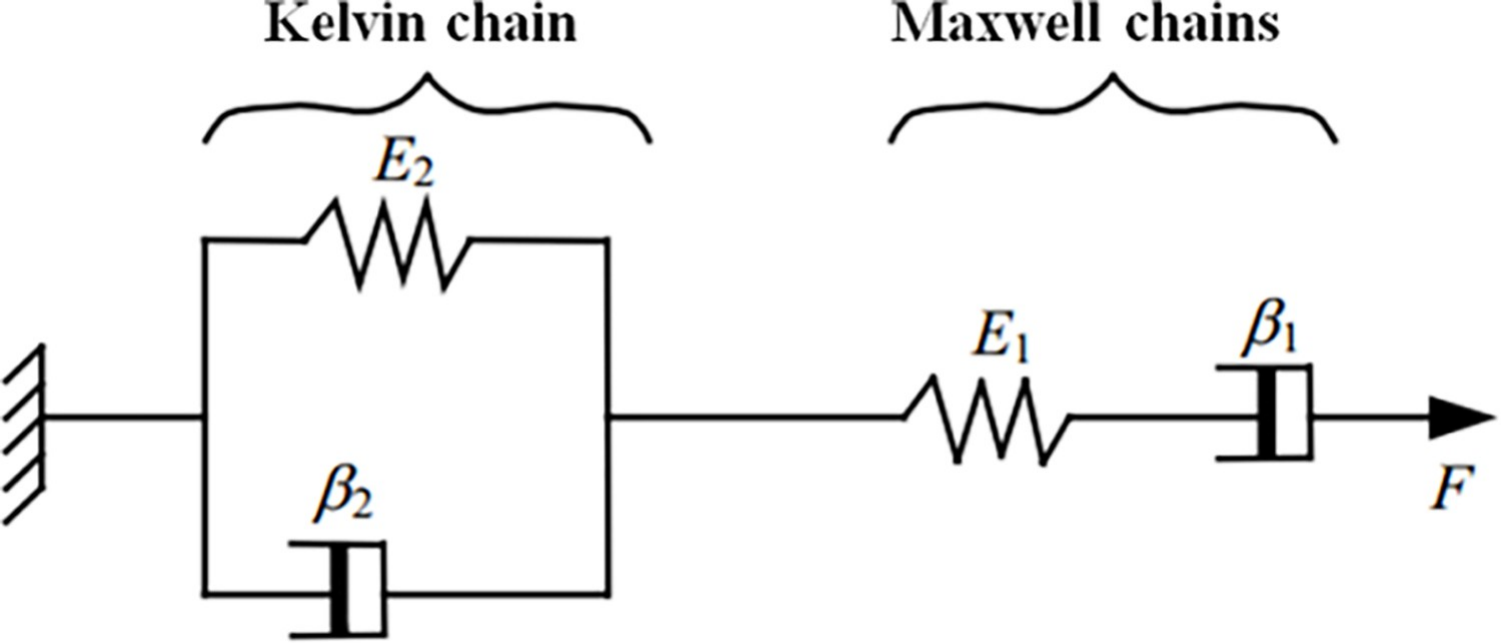
Components of the Burgers model.

At present, there are several methods used to create creep models considering the time effect, e.g., unit-combined methods, empirical formula methods, and yield surface creep models. The above creep test results indicate that the creep curve of the residual soil exhibits obvious instantaneous deformation and that the residual soil primarily exhibits stable creep properties under different stress levels. In view of this, the Burgers model is a suitable choice to describe the creep characteristics of the residual soil [33, 34]. The Burgers model is composed of Kelvin and Maxwell chains in series, as shown in Fig 8. The constitutive relationships of the Burgers model can be described as follows:

$$\sigma +p_{1}\dot{\sigma }+p_{2}\ddot{\sigma }=q_{1}\dot{\varepsilon }+q_{2}\ddot{\varepsilon }$$ (1)

where *p*_1_ = *β*_2_/*E*_2_+*β*_1_/*E*_1_+*β*_1_/*E*_2_, *q*_1_ = *β*_1_, *p*_2_ = *β*_1_*β*_2_/*E*_1_*E*_2_, *q*_2_ = *β*_1_*β*_2_/*E*_2_, and *E*_1_, *E*_2_, *β*_1_, and *β*_2_ are the elastic modulus and the viscosity coefficients of the Maxwell and Kelvin chains, respectively.

The creep equation of the Burgers model can be obtained by solving Eq (1) according to the strain:

$$\varepsilon (t)=\sigma [\frac{1}{E_{1}}+\frac{t}{\beta_{1}}+\frac{1}{E_{2}}(1-e^{-E_{2}t/\beta_{2}})].$$ (2)

It can be seen from Eq (2) that the model can simulate instantaneous deformation, initial creep, and steady creep under loading. It can also simulate instantaneous elastic recovery, elastic hysteresis, and permanent deformation during unloading. However, it cannot simulate the last stage of non-decaying creep, accelerated deformation. For attenuation creep, it is necessary to remove the Newton viscous element in the Maxwell chains, that is, to remove *t*/*β*_1_ in the above formula.

In the shear creep test, for each fixed shear load value *τ*, Eq (2) can be rewritten as follows:

$$\gamma (t)=\tau [\frac{1}{G_{1}}+\frac{t}{\eta_{1}}+\frac{1}{G_{2}}(1-e^{-G_{2}t/\eta_{2}})],$$ (3)

where *G*_1_, *G*_2_, *η*_1_, and *η*_2_ are the shear modulus and viscosity coefficients of the Kelvin and Maxwell chains, respectively.

If parameters apply the following substitutions: *A* = *τ*/*G*_1_, *B* = *τ*/*η*_1_, *C* = *τ*/*G*_2_, and *D* = *G*_2_/*η*_2_, Eq (3) can be further simplified as

$$\gamma (t)=A+Bt+C(1-e^{-Dt}).$$ (4)

When time *t* = 0, the right side of Eq (4) is equal to *A*, that is, *A* can be determined according to the instantaneous deformation. When the time is sufficiently large, the last term *C*(1−*e*^−*Dt*^) on the right side of Eq (4) tends to the constant *C*; then, Eq (4) can be approximated as a linear equation and *B* is the slope of the line. In this way, *B* can be determined by the slope of the final stage of the creep curve. If there is no stable creep stage, *B* is 0. After obtaining *A* and *B*, *C* and *D* can be determined by curve fitting. The values of *C* and *D* can also be determined by the following method. In the decelerated creep stage, two values (*t*_1_, *γ*_1_) and (*t*_2_, *γ*_2_) are taken and substituted into Eq (4). In this way, two equations with *C* and *D* as unknowns can be obtained. The values of *C* and *D* can then be obtained by solving these equations using a numerical method. In this way, all parameters of the Burgers model can be obtained. According to the above method, the Burgers model parameters under different shear forces were obtained, as shown in Tables 3–6.

**Table 3:** Burgers model parameters of the residual soil samples with a rock content of 10%.

*τ*/kPa	*G*_1_/ kPa	*G*_2_/ kPa	*η*_1_/kPa.min	*η*_2_/kPa.min	*R* ^2^
28	0.4E+03	0.3E+03	4.4E+07	0.8E+03	0.9725
56	0.3E+03	0.4E+03	1.0E+06	1.1E+03	0.93495
84	1.1E+03	0.4E+04	1.8E+07	0.2E+03	0.92624
112	0.9E+03	3.7E+03	1.0E+07	5.9E+03	0.99123

**Table 4:** Burgers model parameters of the residual soil samples with a rock content of 30%.

*τ*/kPa	*G*_1_/ kPa	*G*_2_/ kPa	*η*_1_/kPa.min	*η*_2_/kPa.min	*R* ^2^
32	0.9E+03	1.2E+03	3.3E+07	3.1E+03	0.96975
64	0.7E+03	0.5E+03	1.2E+07	1.2E+03	0.93495
96	0.5E+03	0.4E+03	1.9E+07	0.3E+03	0.88911
128	0.2E+03	0.5E+03	0.3E+07	5.6E+03	0.99123

**Table 5:** Burgers model parameters of the residual soil samples with a rock content of 50%.

*τ*/kPa	*G*_1_/ kPa	*G*_2_/ kPa	*η*_1_/kPa.min	*η*_2_/kPa.min	*R* ^2^
36	4.8E+03	0.9E+03	5.2E+07	1.7E+03	0.96975
72	0.9E+03	1.2E+03	2.3E+07	2.5E+03	0.93591
108	0.3E+03	1.0E+03	1.9E+07	1.8E+03	0.86643
144	0.4E+03	0.6E+03	0.8E+07	0.5E+03	0.98091

**Table 6:** Burgers model parameters of the residual soil samples with a rock content of 70%.

*τ*/kPa	*G*_1_/ kPa	*G*_2_/ kPa	*η*_1_/kPa.min	*η*_2_/kPa.min	*R* ^2^
40	3.2E+03	5.2E+03	5.9E+07	1.2E+03	0.84052
80	2.2E+03	1.5E+03	1.3E+08	2.3E+04	0.99153
120	2.5E+03	0.9E+04	1.1E+07	1.3E+04	0.91887
160	0.6E+03	2.0E+03	0.1E+08	3.7E+03	0.98994

## 5. Determination of the long-term strength

The selection of reasonable design parameters for the shear strength of the residual soil plays an important role in establishing a residual soil creep model and the long-term stable operation of a project. At present, the residual strength is often used as the main design parameter in engineering and the creep characteristics and long-term strength of the residual soil are taken as important bases to evaluate the long-term stability.

A variety of methods have been used to obtain the long-term strength parameters of rock masses, such as the Chen Zongji turning point method, orthogonal method, and vertical line method [35]. These methods have advantages and disadvantages. The basic principles encapsulated in these methods can be summarized as follows: the creep process changes from one stage to another, which is related to a certain amount of stress and the creep time. There must be a long-term shear stress level *τ*_∞_ in the creep process. When the shear stress is less than *τ*_∞_, the residual soil is in the stable creep stage; for larger shear stress, the residual soil enters the accelerated creep stage.

**Fig 9:**
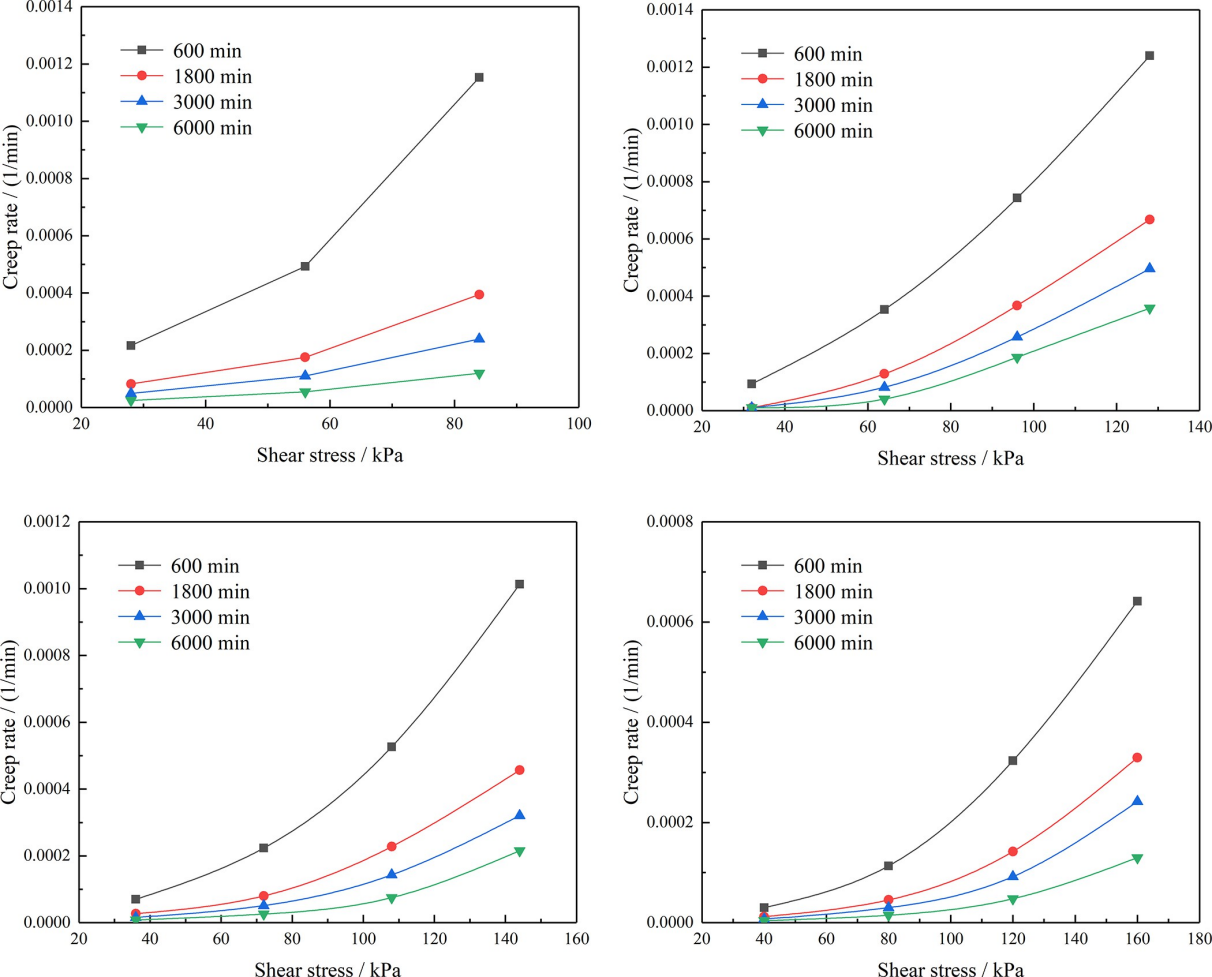
Creep rate–shear stress curves of the residual soil samples. (a) RC = 10%, (b) RC = 30%, (c) RC = 50%, (d) RC = 70%.

After sorting out the creep test data of the residual soil and establishing its isochronous stress–strain curve, it can be observed that the inflection point of the curve is not obvious; therefore, the isochronous curve method was not used. The strain rate isochronous curve method is suitable for a creep test without an inflection point of the isochronous stress–strain curve. Therefore, it can be used the strain rate isochronous curve method to solve for the long-term strength of the residual soil. The four groups of samples with different RCs under the same consolidation pressure were used to obtain the creep rate under different stresses at different times, and the creep rate–stress isochronous curve was established. Because the samples with 10% RC were rapidly shear damaged under the fourth-level shear stress, only the creep rates of the first three levels of load could be obtained for these samples. The creep rate–shear stress curves of the samples with different RCs are shown in Fig 9.

The creep rate is the creep variable per unit time. Firstly, a reference time point *t*_1_ can be selected to determine its corresponding creep variable *ε*_1_, and then the time point *t*_2_ and its corresponding creep parameters *ε*_2_ can be selected. Then, take the difference between the creep variables at two time points to obtain the total amount of creep deformation Δ*ε* between time points. Finally, the creep rate $\Delta \dot{\varepsilon }$ is calculated by dividing the difference Δ*ε* in deformation between time points by the time difference Δ*t*.


$$\Delta \dot{\varepsilon }=\frac{\Delta \varepsilon }{\Delta t}$$ (5)

**Fig 10:**
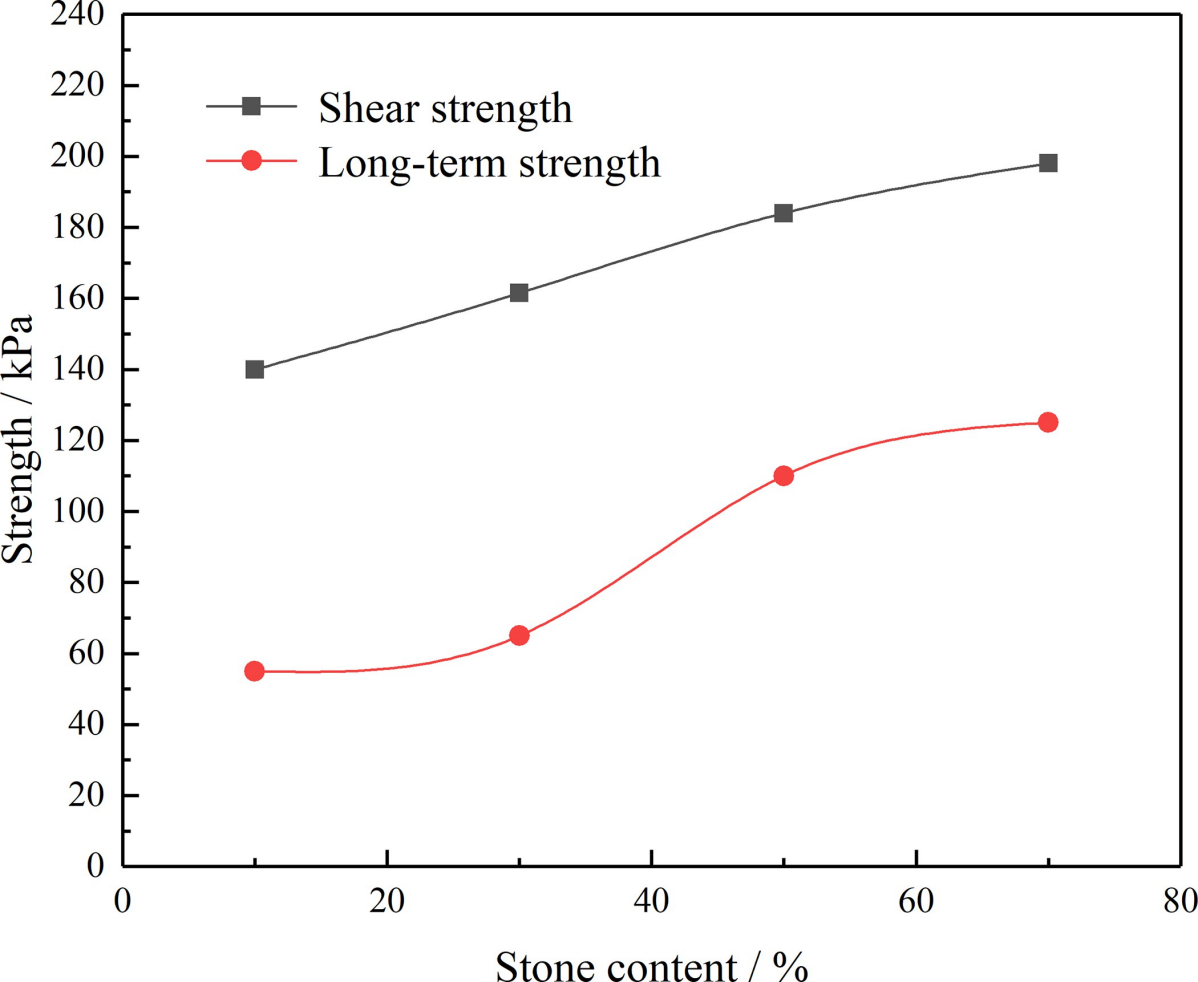
Long-term strength of the residual soil samples with different RCs.

The creep rate is an important index that reflects the deformation rate in the creep test of the residual soil, which is of great significance for the prevention and treatment of landslides in practical engineering. According to the long-term failure criterion theory of landslides, it can be known that one method to maintain the long-term stability of landslides in the later stage is to reduce the creep rate of the landslide rock and soil mass. In this paper, four groups of samples with different RCs (10%, 30%, 50%, and 70%) were configured for shear creep tests. The test results show that the RC has a significant influence on the creep rate of the residual soil. With increasing RC, the creep rate of the residual soil gradually decreases. At the same time, it can be seen from Fig 9 that the creep rate–shear stress curves of the residual soil samples with different RCs have inflection points and that the creep rate before the inflection points is small while the creep rate after the inflection points becomes large and increases rapidly. The shear stress corresponding to the inflection point is the long-term strength of the residual soil. The long-term strengths of the residual soil samples with different RCs are shown in Table 7 and Fig 10.

**Table 7:** Long-term strength of the residual soil samples.

Consolidation pressure /kPa	Rock content/%	Shear strength /kPa	Long-term strength/kPa
200	10	140	55
	30	161.5	65
	50	184	110
	70	198	125

It can be seen from the test results that the RC not only affects the shear strength of the residual soil but also has a significant influence on the long-term strength of the residual soil. With increasing RC, the long-term strength of the residual soil increases nonlinearly. When the RC of the residual soil increases from 10% to 30%, the long-term strength increases from 55 kPa to 65 kPa and the long-term strength change is small. Meanwhile, when the RC increases from 30% to 50%, the long-term strength of the residual soil increases from 65 kPa to 110 kPa, which is a more significant increase. This explains why, given the same slope, soil slopes are more prone to instability failure than soil–rock mixture slopes. At the same time, the long-term strength of residual soil samples with different RCs is far lower than their shear strength and, with increasing RC, the gap between the two becomes smaller. The long-term strength of a residual soil with a RC of 10%–70% is approximately 39.3%–63.1% of its shear strength.

## 6. Conclusions

Taking the residual slope soil of landslides in western Henan as the research object, through the indoor shear creep test of residual slope soil under different rock contents, the influence law of rock content on the creep characteristics and long-term strength of residual slope soil was systematically studied in depth. The conclusions are as follows:

1. The creep style of the residual soil samples with different RCs is attenuated creep. With increasing RC, the instantaneous deformation of the residual soil decreases gradually after applying various shear stresses. After processing the graded loading data of the residual soil using the Boltzmann superposition principle, it was found that the shear creep increases with increasing shear stress. In addition, the RC has a significant influence on the creep deformation of the residual soil. With increasing RC, the creep deformation of the residual soil decreases.
2. The loading time has a significant impact on the stress-strain curve of residual slope soil. With increasing time, the slope of the isochronous stress–strain curves of the residual soil samples with different RCs gradually increases, indicating that the creep increment produced by the soil within the same creep time is increasing.
3. It can be seen from the shear creep tests of the residual soil samples with different RCs that the residual soil exhibits obvious rheological characteristics. Therefore, the Burgers model was used to simulate the rheological process of the residual soil.
4. The RC has a significant impact on the shear strength and long-term strength of the residual soil. With increasing RC, the shear strength and long-term strength of the residual soil gradually increase. At the same time, by comparing the shear strength and the long-term strength of the residual soil samples with different RCs, it was found that the long-term strength is approximately 39.3% to 63.1% of the shear strength.

## Abbreviations

*E*_1_: the elastic modulus of the Maxwell chain, MPa
*E*_1_: the elastic modulus of the Kelvin chain, MPa
*β*_1_: the viscosity coefficients of the Maxwell chain
*β*_2_: the viscosity coefficients of the Kelvin chain
*G*_1_: the shear modulus of the Kelvin chain, MPa
*G*_2_: the shear modulus of the Maxwell chain, MPa
*η*_1_: the viscosity coefficients of the Kelvin chain
*η*_2_: the viscosity coefficients of the Maxwell chain
τ: shear load value, kPa
*τ*_∞_: long-term shear stress level
*ε*_1_: creep variable
